# The Effectiveness of “Two-Week” Referrals for Suspected Bone and Soft Tissue Sarcoma

**DOI:** 10.1155/2007/23870

**Published:** 2008-01-22

**Authors:** A. Malik, L. Wigney, S. Murray, C. H. Gerrand

**Affiliations:** North of England Bone and Soft Tissue Tumour Service, Freeman Hospital, Newcastle upon Tyne NE7 7DN, UK

## Abstract

The two-week “wait” target introduced in 2000 requires that patients with suspected cancer referred by general practitioners should be seen within two weeks. We reviewed patients who had been referred under this standard to the North of England Bone and Soft Tissue Tumour Service, to determine if the referral guidelines had been followed, and what proportion of patients referred under the guideline had malignant tumours. 40 patients were referred under the guideline between January 2004 and December 2005. Ten of these patients (2548%) had malignant tumours, compared with 243 of 507 (48%) of those referred from other sources. In 9 of the 40 cases, the patient did not meet the criteria for urgent referral. Although this target has focussed attention on shortening the time to diagnosis and treatment, prioritising patients referred from general practitioners has the potential to disadvantage those with malignant tumours referred from other sources.

## 1. INTRODUCTION

The NHS Cancer Plan [1] was published in September 2000. This document detailed the government's comprehensive national programme for investment in and reform of cancer services in the NHS. Amongst other reforms, there has been a drive to reduce the waiting time of cancer patients from referral to diagnosis and treatment in a stepwise manner. A maximum two-week wait target for an outpatient appointment for patients with suspected breast cancer has been in place since April 1999. This has been sequentially rolled out to other tumour types. Sarcomas were included in the plan in December 2000.

In December 2005 the next target was introduced; a maximum of 62 days (31 days for children) should elapse between an urgent referral and the initiation of treatment defined as the first diagnostic investigation or treatment intervention. The final target, to be achieved by 2008, is that there should be a maximum wait of 31 days between urgent referral and the initiation of treatment.

Department of Health (DoH) guidelines state that if a soft tissue mass is greater than 5 centimetres in maximum dimension, is deep to the investing fascia, is painful, is being enlarging, or has recurred after previous excision, the patient should be referred under the “two-week” rule [2]. Patients with suspected bone tumours should also be referred under this rule if there are abnormal x-ray findings [2].

As a tertiary referral centre for bone and soft tissue tumours, we receive referrals from a wide variety of sources, including primary care and other hospital consultants. The primary aim of this study was to determine what proportion of new referrals had been made to our centre under the “two-week” rule and what proportion of these patients had malignant tumours. Secondary aims were to determine whether patients being referred under this rule met the referral guidelines, and whether targets for the time from referral to being seen in outpatients and from referral to first diagnostic investigation or treatment were being met.

## 2. METHODS

We reviewed the case notes of all patients referred under the “two-week” rule to the North of England Bone and Soft Tissue Tumour Service between January 2004 and December 2005. The referral letters were evaluated to see if they met DoH guidelines for referral of a suspected bone or soft tissue tumour [2]. Case notes were used to determine the following: the final diagnosis, time elapsed between the referral and first clinic appointment, and the time elapsed between the referral and the first diagnostic investigation or treatment. The total number of referrals to the unit and their diagnosis during this two-year period was obtained from a computerised database.

## 3. RESULTS

Between January 2004 and December 2005, a total of 40 patients were seen under the “two-week” rule. During the same time period, 507 patients were referred by other routes. The final diagnoses for each group are shown in Table 1.

The frequency with which patients had the features described in the guidelines mentioned in the referral letters and the frequency with which these features were detected in the clinic are shown in Table 2. Most (31 of 40, 78%) “two-week” referrals met the published referral guidelines. However, nine referrals under the rule did not mention any of the guideline features. None of these nine patients had malignant tumours. All of the malignant tumours referred under the rule had at least one of the features in the guidelines requiring urgent referral. Eight (20%) of the “two-week” referrals were prompted by suspicious radiological findings, reported by nonspecialist radiologists. Two of these patients had osteochondromata and six had nonneoplastic lesions (stress fracture, cortical sclerosis, medial meniscal cyst, or phlebolith).

A number of 38 of the 40 (95%) patients referred under the “two-week” rule were seen within 14 days (1 to 20 days) following receipt of the referral letter. There was a delay in receiving the referral for the two patients that were seen beyond the two-week target. The median time between referral and first investigation or initiation of treatment for this group was 39 days (range 4 to 358 days). 27 of these 40 (68%) referrals had their treatment initiated or first investigation performed within 62 days of referral. All of the patients with malignant tumours in this group had treatment initiated within 30 days of referral.

## 4. DISCUSSION

Our study shows that the proportion of patients referred to our centre under the “two-week” rule is small (less than a twelfth of the total referrals to the unit). Similarly, the proportion of patients referred under this rule who have malignant tumours is low (6 of 40, 15%). The majority of patients referred to our centre come by other routes, and the proportion of patients with malignant tumours referred in this manner is higher (177 of 507, 35%). 9 of 40 (23%) of the referrals made under the “two-week” rule failed to mention any of the DoH guideline requirements for urgent referral; none of these patients had malignant tumours. Almost all patients referred under the “two-week” rule were seen within two weeks. All patients with malignant tumours were treated or had their first investigation within 30 days of referral. However, overall only 27 of the 40 (68%) patients referred under the “two-week” rule had their treatment initiated or their first investigation performed within 62 days of referral.

Bone and soft tissue sarcomas are rare tumours. Enzinger and Weiss [3] suggested that there are at least 100 benign soft tissue tumours for every malignant tumour examined by a pathologist. The incidence of benign soft tissue tumors is about 300 per 100,000 population [4]. A general practitioner (GP) with a practice of 3000 patients might expect to see at least three patients with benign soft tissue tumors every year. However, the same GP would only expect to see one patient with a soft tissue sarcoma every 24 years [5]. Diagnosing a malignant soft tissue tumor requires a high index of suspicion. The “two-week” target was clearly intended to raise awareness of these tumours amongst the primary care team and to shorten the wait for a patient with a suspicious mass to be seen in a specialist clinic and therefore reduce the delays in treatment. This is a laudable ambition. However, the imposition of this kind of target for a selected group of patients (those thought by a GP to be a priority) has the potential to interfere with the delivery of rapid care to the remainder [6]. It is important that patients with sarcomas referred by other routes are not disadvantaged by the prioritisation of patients identified as urgent under the “two-week” rule, particularly when the referral guidelines have not been followed. We would like all patients with sarcoma to be seen and treated in as short a time as possible, regardless of how they are referred.

This was a retrospective study, limited by the data available. We were therefore unable to identify particular instances in which patients referred with benign conditions under the “two-week” rule had been prioritised over patients with sarcomas referred by other routes. However, the potential for this remains, particularly if the volume of cases referred under the “two-week” rule increases. We believe that clinicians working in a service are in the best position to distribute access to limited resources according to clinical need.

## Figures and Tables

**Table 1:** Distribution of diagnostic categories for each referral route.

Route of referral	Primary tumour malignant	Primary tumour benign	Metastatic tumour	Nonneoplastic	Total
“Two-week”rule	6 (15%)	12 (30.5%)	4 (7.5%)	18 (46%)	40
Other	175 (35%)	220 (43%)	68 (13%)	44 (8%)	507

**Table 2:** Frequency with which clinical features in DoH referral guidelines appeared in referral letters and after review in our centre.

Feature	Frequency in letter	Frequency in clinic
Size *>* 5 cms	24 (60%)	20 (50%)
Pain	22 (55%)	14 (35%)
Increase insize	19 (47.5%)	18 (45%)
Deep tofascia	32 (80%)	26 (65%)
Recurrence	0 (0%)	0 (0%)
SuspiciousX-rays	8 (20%)	8 (20%)
None	9 (22.5%)	7 (17.5%)
